# Supplementary material for: Chromothripsis during telomere crisis is independent of NHEJ, and consistent with a replicative origin
Source: Genome Res. 2019 May;29(5):737–49. doi: 10.1101/gr.240705.118 (PMC6499312; doi:10.1101/gr.240705.118)
Supplement: Supplemental Material [file supp_gr.240705.118_Supplemental_file_1.zip › contigs/annotated_contigs/DB53/contig.2.DB53_length_464_mean_cov_5.23275862069.docx]

**DB53_length_464_mean_cov_5.23275862069**

```
TGAAATCAAAGCATCACGATTGAGCCCAGAACATGAAAAAGAACTTCCTGGCCCACTTGAG|A|CCCCGTCTCCACTAAAAAATACAAA
 >chr8:133135759-133135821 - E=1e-25                          >chr8:133473634-133474036
AACTAGCCGGGCGTGGTGGTGGGCACCTGTAATCCTAGCTACTCAGGAGGCTGAGGCAGGAGAATTGCTTGAACCCAGGAGGTGAAGGT
 + E=1e-203
TGCAGTGAGCCAAGATTGTGCCATTGCACTCCAGCCTGGGCGACAAGAGCAGGACTCCGTCTCAAAAAAAAGAAAAGAAAAGAAAAGGC

ACCCACTGCCTTCCAGGGTGCCTGTACCATTTTTCCATTCCCATCAGCAATGAATCACAGTTCCTGTTGCCCTACATCCTTGTCAACAT

TTGACATTGTCCTCGCTTTGGATTTTAGCCACCCCCATTCAAATAGGTTTGTAGTGAGATCTCATTTGGGTTTTAATTTACAAATCCCT

GACGAAATGATATTGAGCAT|A
```
